# Pretreatment with laccase and a phenolic mediator degrades lignin and enhances saccharification of *Eucalyptus* feedstock

**DOI:** 10.1186/1754-6834-7-6

**Published:** 2014-01-08

**Authors:** Alejandro Rico, Jorge Rencoret, José C del Río, Angel T Martínez, Ana Gutiérrez

**Affiliations:** 1 Instituto de Recursos Naturales y Agrobiología de Sevilla, CSIC, Reina Mercedes, 10, E-41012 Seville, Spain; 2 Centro de Investigaciones Biológicas, CSIC, Ramiro de Maeztu 9, E-28040 Madrid, Spain

**Keywords:** 2D NMR, Analytical pyrolysis, Bioethanol, *Eucalyptus globulus*, Enzymatic delignification, Laccase, Lignin, Lignocellulose, Pretreatment, Saccharification

## Abstract

**Background:**

Biofuel production from lignocellulosic material is hampered by biomass recalcitrance towards enzymatic hydrolysis due to the compact architecture of the plant cell wall and the presence of lignin. The purpose of this work is to study the ability of an industrially available laccase-mediator system to modify and remove lignin during pretreatment of wood (*Eucalyptus globulus*) feedstock, thus improving saccharification, and to analyze the chemical modifications produced in the whole material and especially in the recalcitrant lignin moiety.

**Results:**

Up to 50% lignin removal from ground eucalypt wood was attained by pretreatment with recombinant *Myceliophthora thermophila* laccase and methyl syringate as mediator, followed by alkaline peroxide extraction in a multistage sequence. The lignin removal directly correlated with increases (approximately 40%) in glucose and xylose yields after enzymatic hydrolysis. The pretreatment using laccase alone (without mediator) removed up to 20% of lignin from eucalypt wood. Pyrolysis-gas chromatography/mass spectrometry of the pretreated wood revealed modifications of the lignin polymer, as shown by lignin markers with shortened side chains and increased syringyl-to-guaiacyl ratio. Additional information on the chemical modifications produced was obtained by two-dimensional nuclear magnetic resonance of the whole wood swollen in dimethylsulfoxide-*d*_6_. The spectra obtained revealed the removal of guaiacyl and syringyl lignin units, although with a preferential removal of the former, and the lower number of aliphatic side-chains per phenylpropane unit (involved in main β-*O*-4ʹ and β-βʹ inter-unit linkages), in agreement with the pyrolysis-gas chromatography/mass spectrometry results, without a substantial change in the wood polysaccharide signals. However, the most noticeable modification observed in the spectra was the formation of C_α_-oxidized syringyl lignin units during the enzymatic treatment. Further insight into the modifications of lignin structure, affecting other inter-unit linkages and oxidized structures, was attained by nuclear magnetic resonance of the lignins isolated from the eucalypt feedstock after the enzymatic pretreatments.

**Conclusions:**

This work shows the potential of an oxidative enzymatic pretreatment to delignify and improve cellulase saccharification of a hardwood feedstock (eucalypt wood) when applied directly on the ground lignocellulosic material, and reveals the main chemical changes in the pretreated material, and its recalcitrant lignin moiety, behind the above results.

## Background

Lignocellulosic biomass is a renewable resource of great interest for the sustainable production of fuels, materials and chemicals. Among biomass sources, *Eucalyptus* plantations offer a viable feedstock because they are among the fastest growing tree plantations in the world [1]. However, the conversion of lignocellulosic biomass is challenged by its recalcitrant structure. Cellulose, hemicelluloses and lignin are the three main components of lignocellulose, linked into a complex matrix highly resistant to chemical and biological conversion. Biofuel production from lignocellulosic material requires deconstruction of the cell wall into individual polymers, and hydrolysis of the carbohydrates into monomeric sugars. One of the major factors causing biomass recalcitrance towards saccharification is correlated with the content and composition of lignin [2-4].

Lignin is a three-dimensional polymer constituted by phenylpropanoid subunits linked together by a variety of ether and carbon-carbon bonds. Lignin is intimately interlaced with hemicelluloses in the plant cell wall forming a matrix to cover the crystalline cellulose microfibrils. Its aromatic nature and complex structure make lignin degradation very difficult. Both lignin and lignin-derived compounds have a detrimental effect on the hydrolysis of biomass because they physically hinder the accessibility of cellulases; they also bind cellulases and lead to their inactivation [5-9]. Biotechnology can contribute to plant biomass deconstruction by providing biocatalysts to degrade or modify lignin and lignin-derived compounds [10].

Biomass pretreatment to remove lignin is essential for the enzymatic hydrolysis of lignocellulose. Physical, chemical and biological pretreatments, or combinations of these processes, are being studied for deconstructing lignocellulosic biomass and removing lignin [11-13]. Most biological pretreatments employ lignin-degrading fungi belonging to the group of white-rot basidiomycetes [14,15] but such pretreatments require long application periods and consume a fraction of the plant polysaccharides.

Laccases (phenoloxidases, EC 1.10.3.2) are multicopper oxidases that oxidize substituted phenols using molecular oxygen as the final electron acceptor. The direct action of laccases on lignin is, in principle, restricted to phenolic units, which only represent a small percentage of the total polymer, a fact that limits their biotechnological application. However, the discovery that some synthetic compounds can act as electron carriers between the enzyme and the final substrate [16], 1-hydroxybenzotriazole (HBT) being among the most efficient ones [17], has expanded the utility of laccases. A number of studies have confirmed the potential of laccase-mediator systems for paper pulp delignification [18,19], pitch control [20], polymer modification [21], other applications in the forest industry [22], and bioethanol production from physically and/or chemically pretreated lignocellulose [23]. Recently, the ability of high redox-potential laccases from basidiomycetes of the genus *Trametes* to remove lignin (when applied in combination with HBT) from whole [24] and ensiled [25] lignocellulosic biomass, making cellulose accessible to hydrolysis, was reported. However, most of the studied mediators are synthetic compounds based on nitrogen heterocycles whose high cost and potential toxicity make it difficult to implement laccase-mediator systems at an industrial scale.

Recently, several natural phenols, which form stable aromatic radicals and are available as chemical pulping by-products [26], have been investigated as laccase mediators for pulp biobleaching [27-29] and removal of lipophilic extractives from paper pulp [26]. In the present study, a recombinant laccase from the ascomycete *Myceliophthora thermophila* in combination with the natural mediator methyl syringate was tested for the removal of lignin from *Eucalyptus globulus* wood feedstock. The modification of lignin in the pretreated lignocellulosic material was analyzed by pyrolysis coupled to gas chromatography/mass spectrometry (Py-GC/MS) and two-dimensional nuclear magnetic resonance (2D NMR) spectroscopy of the whole sample at the gel state [30,31]. Additionally, lignin was isolated from the pretreated samples and further characterized by 2D NMR. In addition to lignin modification and removal, the effect of the laccase-mediator on the saccharification yield from the pretreated eucalypt feedstock was assessed.

## Results

### Delignification of eucalypt wood by laccase with and without methyl syringate

Two doses of *M. thermophila* laccase (10 U · g^-1^ and 50 U · g^-1^) [24] and methyl syringate (1% and 3%) were tested in the enzymatic pretreatment of eucalypt wood feedstock. This consisted of a sequence of four laccase-mediator treatments, each followed by an alkaline peroxide extraction step. The lignin contents of eucalypt samples after the whole laccase-mediator sequence were determined (as Klason lignin) and compared with their respective controls (Table 1). The amount of lignin decreased considerably after the enzymatic sequence, concomitantly with increasing laccase doses. The decreases were about 37% and 47% of the initial lignin content when using laccase doses of 10 U · g^-1^ and 50 U · g^-1^ in combination with 1% and 3% methyl syringate, respectively. The treatments with laccase alone (without mediator) decreased the lignin content about 12% and 20% when using laccase doses of 10 U · g^-1^ and 50 U · g^-1^, respectively.

**Table 1:** **Lignin content and monosaccharides release** (% **of sample weight**) **by cellulase hydrolysis of eucalypt samples**

**Eucalypt samples**	**Lignin (%)**	**Glucose (%)**	**Xylose (%)**
Initial eucalypt wood	22.3 ±0.3	39.5 ±1.1	6.7 ±0.1
Control	21.1 ±1.0	43.7 ±0.2	7.5 ±0.1
Laccase (10 U · g^-1^)-MeS (1%)	13.3 ±0.1	54.8 ±1.0	9.2 ±0.2
Laccase (50 U · g^-1^)-MeS (3%)	11.2 ±0.3	55.7 ±0.4	9.1 ±0.1
Laccase (10 U · g^-1^)	18.5 ±0.4	46.3 ±0.8	7.6 ±0.1
Laccase (50 U · g^-1^)	16.8 ±0.3	47.8 ±1.2	8.1 ±0.2

Lignin content (as Klason lignin) and monosaccharides from cellulase hydrolysis of eucalypt samples treated with *M. thermophila* laccase (10 U · g^-1^ and 50 U · g^-1^) and methyl syringate (MeS) mediator (1% and 3%) in a sequence including four enzymatic treatments (and four alkaline peroxide extractions) compared with a control without enzyme, a treatment with laccase alone, and the initial eucalypt wood. Means ± SD (from triplicates).

### Enzymatic hydrolysis of pretreated eucalypt wood

The wood samples treated with laccase (10 U · g^-1^ and 50 U · g^-1^), alone and in the presence of methyl syringate (1% and 3%, respectively), as well as the corresponding controls (and the initial untreated wood) were hydrolyzed (72 h) using a cellulase and β-glucosidase cocktail [24], and the main monosaccharides released (glucose and xylose) were analyzed by GC. When low cellulase (2 filter-paper units [FPU] · g^-1^) and β-glucosidase (100 nkat · g^-1^) doses were used, increases in glucose yields up to 39% and 41% (with respect to the initial eucalypt wood sample) were attained in the samples pretreated with 10 U · g^-1^ and 50 U · g^-1^ of laccase, in combination with 1% and 3% mediator, respectively (Table 1). In the samples pretreated with 10 U · g^-1^ and 50 U · g^-1^ of laccase alone (without mediator), increases in glucose release of 17% and 21%, respectively, were produced. The effect of oxygen and alkaline extraction steps in the control sample were responsible for the increase of 11% in glucose yield with respect to the initial eucalypt sample. An improvement on xylose release of about 37% was obtained after the laccase-mediator treatment of eucalypt wood (with respect to the initial eucalypt wood sample). This improvement was similar with the two different doses of laccase-mediator used. However, in the pretreatment with laccase alone, different increases in xylose yields (13% and 21%) were obtained with the two laccase doses (10 U · g^-1^ and 50 U · g^-1^, respectively). The effect of oxygen and alkaline extraction on xylose yield (control sample with respect to the initial one) represented an increase of 12%.

### Pyrolysis coupled to gas chromatography/mass spectrometry of pretreated eucalypt wood

Modification of the eucalypt lignin by the enzymatic pretreatment was studied by Py-GC/MS. This degradative technique allows for *in situ* analysis of lignin by chromatographic separation and mass-spectrometric identification of the compounds released after the pyrolytic breakdown of whole wood samples (Table 2). The main lignin-derived compounds (lignin markers) released were guaiacol, 4-methylguaiacol, 4-ethylguaiacol, 4-vinylguaiacol, syringol, 4-methylsyringol, *trans*-isoeugenol, 4-ethylsyringol, 4-vinylsyringol, 4-allylsyringol, *cis*-4-propenylsyringol, syringaldehyde, *trans*-4-propenylsyringol and *trans*-sinapaldehyde.

**Table 2:** **Relative molar abundances of lignin markers from Py**-**GC**/**MS of eucalypt wood treated with laccase-mediator, laccase alone and control**

**Compound**	**Control**	**Laccase only**	**Laccase-mediator**
Guaiacol (G)	4.4	4.8	6.4
4-methylguaiacol (G-CH3)	2.6	2.0	1.2
4-ethylguaiacol (G-CH2-CH3)	1.0	1.2	1.2
4-vinylguaiacol (G-CH = CH2)	4.0	3.5	2.9
Eugenol (G-CH2-CH = CH2)	0.9	0.8	0.5
Syringol (S)	19.2	21.7	32.0
*cis*-isoeugenol (G-CH = CH-CH3)	0.7	0.5	0.3
*trans*-isoeugenol (G-CH = CH-CH3)	4.0	3.6	2.0
4-methylsyringol (S-CH3)	7.5	7.2	5.0
Vanillin (G-CHO)	1.3	1.0	0.7
4-ethylsyringol (S-CH2-CH3)	3.5	3.8	5.3
Acetovanillone (G-CO-CH3)	0.7	0.9	1.1
4-vinylsyringol (S-CH = CH2)	13.3	12.4	12.3
Guaiacylacetone (G-CH2-CO-CH3)	0.5	0.5	0.5
4-allylsyringol (S-CH2-CH = CH2) + 4-propylsyringol (S-CH2-CH2-CH3)	4.3	4.1	3.6
*cis*-propenylsyringol (S-CH = CH-CH3)	2.6	2.3	1.8
*trans*-propenylsyringol (S-CH = CH-CH3)	14.4	12.8	10.2
Syringaldehyde (S-CHO)	5.8	4.5	2.5
Homosyringaldehyde (S-CH2-CHO)	0.0	1.1	0.9
Acetosyringone (S-CO-CH3)	3.6	5.0	4.9
Syringylacetone (S-CH2-CO-CH3)	2.2	2.2	2.3
Propiosyringone (S-CO-CH2-CH3)	0.7	0.8	0.6
Dihydrosinapyl alcohol (S-CH2-CH2-CH2OH)	0.7	0.8	0.5
*trans*-sinapyl alcohol (S-CH = CH-CH2OH)	0.8	0.2	0.3
*trans*-sinapaldehyde (S-CH = CH-CHO)	1.0	2.3	1.0
C_6_-C_0-2_/C_6_-C_3_ ratio	2.0	2.2	3.2
Syringyl-to-guaiacyl ratio	4.0	4.3	4.9

Main lignin-derived compounds (lignin markers) from Py-GC/MS of eucalypt wood treated with *M. thermophila* laccase (50 U · g^-1^) and the mediator methyl syringate (3%) in a sequence including four enzymatic treatments and four alkaline peroxide extractions compared with a control without enzyme, and a treatment with laccase alone. Methyl syringate was also recovered among the Py-GC/MS products from the laccase and methyl syringate sample (amounting to 8% of the listed lignin-derived markers). The ratio between lignin markers with reduced side chains (C_6_-C_0-2_) and phenylpropane (C_6_-C_3_) markers, as well as the syringyl-to-guaiacyl ratio, are also indicated.

The results of the Py-GC/MS analyses of the control wood indicated that the eucalypt lignin was rich in S-units, with a syringyl-to-guaiacyl (S/G) ratio of 4.0 for the control sample. Interestingly, the laccase-mediator treatment caused a decrease in the G-lignin units with respect to the S-lignin ones, resulting in an increase of the S/G ratio to 4.9. The decrease of phenylpropane type pyrolysis compounds was also noticeable, as shown by the ratio between reduced-chain (C_6_-C_0-2_) and full-chain (C_6_-C_3_) lignin markers, which increased from 2.0 (control wood) to 3.2 in the laccase-mediator-treated sample, revealing cleavage of the lignin unit side-chains by the enzymatic pretreatment. Both the side-chain reduction and the S/G ratio increase tendencies were also observed in the eucalypt wood pretreated with laccase alone, although the changes produced were much more moderate than those obtained in the presence of methyl syringate.

### Two-dimensional nuclear magnetic resonance of pretreated eucalypt wood

**Figure 1:**
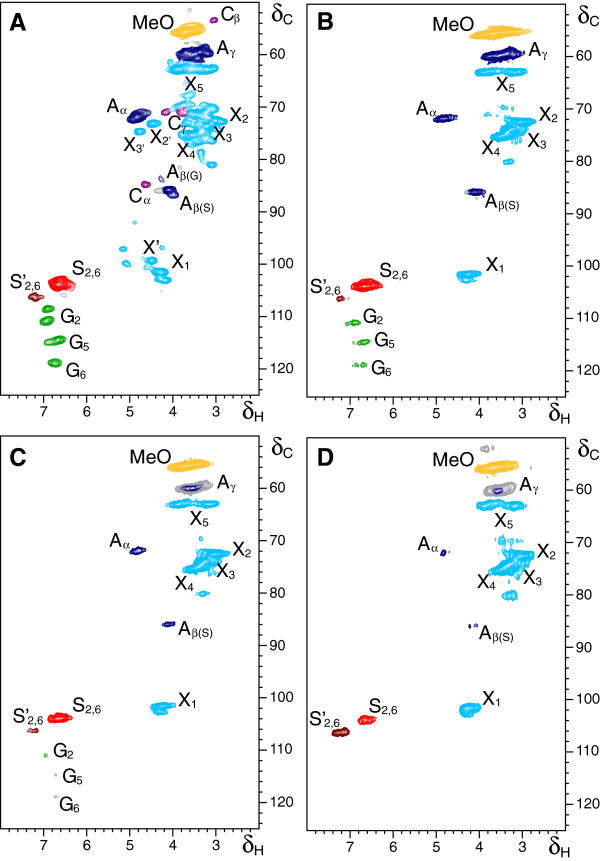
**Heteronuclear single quantum correlation nuclear magnetic resonance spectra of whole eucalypt samples swollen in dimethylsulfoxide**-***d***_**6**_**. (A)** Initial sample, **(B)** control without enzyme, **(C)** sample treated with laccase alone (50 U · g^-1^) and **(D)** sample treated with laccase (50 U · g^-1^) and methyl syringate (3%). See Table 3 for lignin signal assignment, Figure 2 for the main lignin structures identified and Table 4 for quantification of these lignin structures. Correlation signals from normal (X_1_-X_5_) and acetylated xylan (Xʹ_1_-Xʹ_3_) are also indicated. The 52/3.8 ppm signal corresponds to some methyl syringate incorporated onto the lignin (see Figure 3). The enzymatic pretreatment included four laccase-mediator treatments, each followed by an alkaline peroxide extraction step.

**Figure 2:**
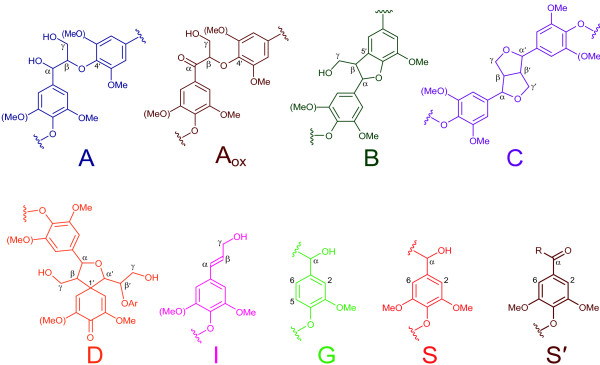
**Main lignin structures identified in the eucalypt samples analyzed by heteronuclear single quantum correlation nuclear magnetic resonance (Figures**1**and**3**).** A = β-*O*-4ʹ lignin substructures (including a second S or G unit); Aox = C_α_-oxidized β-*O*-4ʹ lignin substructures; B = phenylcoumarans; C = resinols; D = spirodienones; I = cinnamyl alcohol end-groups; G = guaiacyl units; S = syringyl units; and Sʹ = C_α_-oxidized S units (R can be a hydroxyl in carboxylic acids or a lignin side-chain in ketones).

The modification of lignin structure after the whole enzymatic pretreatments of eucalypt wood was also studied by 2D NMR. With this purpose, the wood samples were swelled in deuterated dimethylsufoxide (DMSO-*d*_*6*_) forming a gel, and analyzed by heteronuclear single quantum correlation (HSQC) NMR. Figure 1 shows the HSQC spectra of the wood samples before (initial wood), after laccase (alone) and after laccase-mediator treatments with the higher laccase (50 U · g^-1^) and mediator (3%) doses. The control treatment, without enzyme and mediator, was also analyzed. The aliphatic oxygenated region of the spectra shows methoxyl, lignin side-chain and carbohydrate cross-signals, and the aromatic region, include the signals of S- and G-lignin units. The main lignin structures identified are shown in Figure 2, and the different lignin signals assigned on the spectra are listed in Table 3. Table 4 shows the composition of lignin, in terms of S and G units, and the relative abundance of the main inter-unit linkages in the different samples, which were estimated from the signal volume integrals.

**Table 3:** **Assignments of lignin main **^**13**^**C**-^**1**^**H correlation signals in the heteronuclear single quantum correlation spectra of eucalypt wood and lignins**

**Label**	**δ**_**C**_**/δ**_**H **_**(ppm)**	**Assignment**
B_β_	53.1/3.45	C_β_ - H_β_ in phenylcoumaran substructures **(B)**
C_β_	53.3/3.05	C_β_ - H_β_ in β - βʹ resinol substructures **(C)**
MeO	55.6/3.72	C - H in methoxyls
A_γ_	59.4 /3.38 and 3.70	C_γ_ - H_γ_ in β-*O*-4ʹ structures **(A)**
D_β_	59.7/2.73	C_β_ - H_β_ in spirodienone substructures **(D)**
I_γ_	61.3/4.08	C_γ_ - H_γ_ in cinnamyl alcohol end-groups **(I)**
B_γ_	62.6/3.67	C_γ_ - H_γ_ in phenylcoumaran substructures **(B)**
A_α_	71.8/4.85	C_α_ - H_α_ in β-*O*-4ʹ structures **(A)**
C_γ_	71.0/ 3.81 and 4.18	C_γ_ - H_γ_ in β - βʹ resinol substructures **(C)**
D_β'_	79.2/4.10	C_βʹ_ - H_βʹ_ in spirodienone substructures **(D)**
D_α_	81.0/5.08	C_α_ - H_α_ in spirodienone substructures **(D)**
A_oxβ_	83.0/5.20	C_β_ - H_β_ in α-oxidized β-O-4′ substructures **(A**_**ox**_**)**
A_β(G)_	83.6/4.28	C_β_ - H_β_ in β-*O*-4ʹ structures **(A)** linked to a G-unit
D_α'_	83.7/4.68	C_αʹ_ - H_αʹ_ in spirodienone substructures **(D)**
C_α_	84.7/4.64	C_α_ - H_α_ in β - β′ resinol substructures **(C)**
A_β(S)_	85.7/4.10	C_β_ - H_β_ in β-*O*-4ʹ structures (A) linked to a S-unit
B_α_	86.4/5.43	C_α_ - H_α_ in phenylcoumaran substructures **(B)**
S_2,6_	103.9/6.69	C_2_ - H_2_ and C_6_ - H_6_ in syringyl units **(S)**
Sʹ_2,6_	106.1/7.18 and 7.31	C_2_ - H_2_ and C_6_ - H_6_ in α-oxidized syringyl units **(Sʹ)**
G_2_	110.8/6.96	C_2_ - H_2_ in guaiacyl units **(G)**
D_2_	113.3/6.25	C_2_ - H_2_ in spirodienone substructures **(D)**
G_5_	114.3/6.69, 114.9/6.94	C_5_ - H_5_ in guaiacyl units **(G)**
G_6_	118.8/6.78	C_6_ - H_6_ in guaiacyl units **(G)**
D_6_	118.7/6.06	C_6_ - H_6_ in spirodienone substructures **(D)**

**Figure 3:**
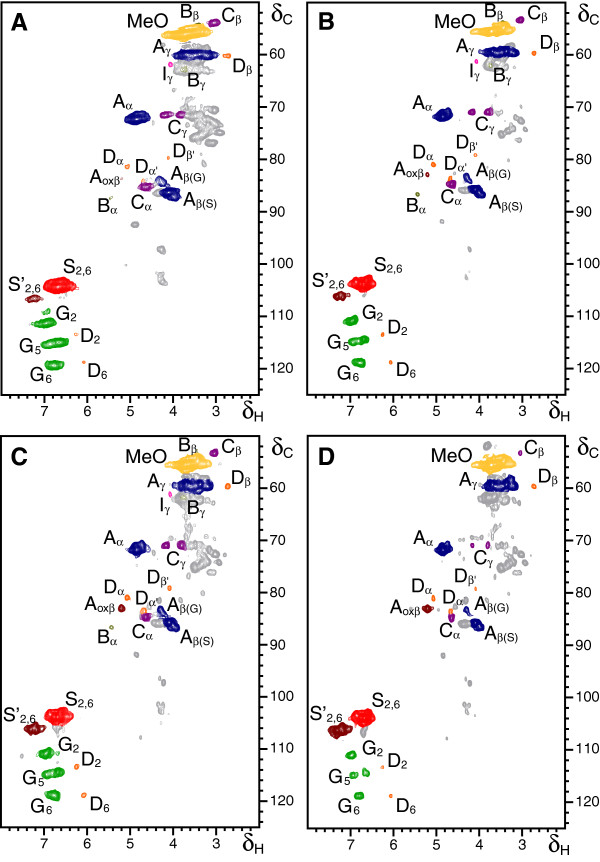
**Heteronuclear single quantum correlation nuclear magnetic resonance spectra of cellulolytic enzyme lignins isolated from eucalypt wood samples. (A)** Initial eucalypt sample, **(B)** control without enzyme, **(C)** sample treated with laccase alone (50 U · g^-1^) and **(D)** sample treated with laccase (50 U · g^-1^) and methyl syringate (3%). See Table 3 for lignin signal assignment, Figure 2 for the main lignin structures identified, and Table 5 for quantification of these lignin structures identified. The 52/3.8 ppm signal corresponds to some methyl syringate incorporated onto the lignin. The enzymatic pretreatment included four laccase-mediator treatments, each followed by an alkaline extraction step.

From heteronuclear single quantum correlation spectra in Figures 1 and 3. See Figure 2 for chemical structures indicated by letters in bold.

**Table 4:** **Lignin units and side-chains forming different inter**-**unit linkages from the heteronuclear single quantum correlation spectra of treated eucalypt wood and controls**

	**Wood**	**Control**	**Laccase only**	**Laccase and methyl syringate**
***Lignin units***				
Syringyl **(S)** (% total)	78	79	86	100
Guaiacyl **(G)** (% total)	22	21	14	0
C_α_-oxidized S units **(Sʹ)** (% S)	13	11	16	47
S/G ratio	3.6	3.8	6.3	-
***Chains forming inter-unit linkages*** (% *S* + *G*)				
β-*O*-4ʹ Alkyl-aryl ethers **(A)**	61 (86)	48 (92)	42 (100)	23 (100)
Phenylcoumarans **(B)**	0	0	0	0
Resinols **(C)**	10 (14)	4 (8)	0	0
Spirodienones **(D)**	0	0	0	0
Total	71 (100)	52 (100)	42 (100)	23 (100)

The lignin composition (S and G units), the amount of C_α_-oxidized S units (with respect to total S units), the S/G ratio, and the abundance of side chains forming different inter-unit linkages (A*-*D) per 100 phenylpropane units were determined from the heteronuclear single quantum spectra of eucalypt wood treated with laccase (50 U · g^-1^) and 3% methyl syringate and laccase alone, compared with a control without enzyme and the initial wood. The percentage of side chains forming each linkage type (or end-group) are also indicated in parentheses.

The aliphatic-oxygenated region of the HSQC spectrum of the initial eucalypt wood (Figure 1A, top-right) shows signals of lignin and carbohydrates, the latter mainly corresponding to xylan (X) and acetylated xylan (X') units, since crystalline cellulose is nearly 'silent' in lignocellulose gel spectra under solution NMR conditions. In this region, signals of side-chains in β-*O*-4' alkyl-aryl ether lignin substructures (A), including C_γ_-H_γ_, C_β_-H_β_ and C_α_-H_α_ correlations (A_γ_, A_β_ and A_α_, respectively) were observed. The A_γ_ signal overlapped with related signals in lignin and other lignocellulose constituents. The C_β_-H_β_ correlations gave two different signals corresponding to β-*O*-4' substructures where the second unit was an S unit or a G unit (A_β(S)_ and A_β(G)_), the latter with lower intensity, in agreement with lignin composition described below. Other less prominent signals for resinol (β–β') substructures (C) were also observed in the spectrum, with their C_α_–H_α_, C_β_–H_β_ and the double C_γ_–H_γ_ correlations (C_α_, C_β_ and C_γ_). The main signals in the aromatic region of the HSQC spectrum (Figure 1A, bottom-left) corresponded to the benzenic rings of the S and G lignin units. The S-lignin units showed a prominent signal for the C_2,6_-H_2,6_ correlation (S_2,6_), whereas the G-lignin units showed different correlations for C_2_-H_2_ (G_2_), C_5_-H_5_ (G_5_) and C_6_-H_6_ (G_6_). Signals corresponding to C_2,6_-H_2,6_ correlations in C_α_-oxidized S-lignin units (S'_2,6_) were also observed although in low quantities. From the integrals of the above signals, an S/G ratio around 3.6, and a large predominance of β-*O*-4' ether linkages together with some resinols, was estimated for lignin in *E. globulus* wood (Table 4).

The HSQC spectrum of the eucalypt control sample at the end of the whole sequence (Figure 1B) showed some differences compared to the initial eucalypt. The most remarkable was the disappearance of the signals of the acetylated xylan units (X'). This may have been caused by the conditions of the control treatment (oxygen addition and alkaline peroxide extractions) that was performed under the same conditions as the enzyme-pretreated samples except for the addition of enzyme and mediator. Concerning lignin side chains, the spectrum of the control sample also revealed some decrease in the amount of β-*O*-4' alkyl-aryl ethers (about 21%) and resinol (about 60%) substructures per 100 phenylpropane units, with zrespect to the initial wood (Table 4). Less intense signals of G and S'_2,6_ units than in the initial eucalypt wood were also observed in the aromatic region.

The HSQC spectrum of the eucalypt sample treated with laccase-mediator at the end of the whole sequence (Figure 1D) showed important differences compared to the control. The signals of side-chains in β-*O*-4' lignin substructures (A_α_ and A_β(S)_) decreased considerably with respect to the carbohydrate and S-lignin signals. The G lignin signals completely disappeared with the laccase-mediator treatment, whereas the S units were C_α_-oxidized (and in a significant extent remained as such), as revealed by the increase in the S'_2,6_ signal. The results obtained showed a C_α_-oxidation mechanism for lignin removal by laccase in the presence of methyl syringate, and revealed that about half of the residual lignin in the laccase-mediator-treated wood corresponded to the C_α_-oxidized S units, as shown in Table 4 where the contribution of methyl syringate to the 106/7.3 ppm signal was subtracted. Finally, the low intensity of the aromatic and aliphatic-oxygenated lignin signals in the HSQC spectrum of the laccase-mediator-treated sample, compared to the carbohydrate signals, was in agreement with the reduced Klason lignin content (Table 1).

Interestingly, lignin modification and removal was also shown by the NMR spectra of the eucalypt feedstock treated with laccase alone (Figure 1C), with a relative decrease of the lignin signals compared to the carbohydrate signals, although not as evident as that observed after the laccase-mediator treatment. Among them, the signals of side-chains in β-*O*-4' lignin substructures (A_α_ and A_β(S)_) and especially the G lignin signals, decreased considerably with respect to the control sample (Table 4), although the changes were less intense than those found in the sample treated with laccase and methyl syringate. Likewise, the C_α_-oxidation of S units was much less pronounced than found in the presence of methyl syringate.

### Two-dimensional nuclear magnetic resonance of lignin isolated from pretreated eucalypt wood

To gain further insight into the modification of lignin structure with the laccase-mediator treatment, cellulolytic enzyme lignin (CEL) was isolated from the pretreated eucalypt samples, and analyzed by 2D NMR (Figure 3). The lignin structures identified, some of them not detected in the wood spectra, are shown in Figure 2, and the corresponding signals are listed in Table 3. Table 5 shows the lignin composition, and percentages of inter-unit linkages (and end-groups) in the different CEL samples, estimated from the signal volume integrals as described for the wood spectra.

**Table 5:** **Lignin units and side-chains forming different inter**-**unit linkages** (**and end**-**groups**) **from the heteronuclear single quantum correlation spectra of cellulolytic enzyme lignin preparations from treated wood and controls**

**Lignin structure**	**Wood**	**Control**	**Laccase only**	**Laccase and methyl syringate**
***Lignin units***				
Syringyl **(S)** (% total)	77	81	83	93
Guaiacyl **(G)** (% total)	23	19	17	7
C_α_-oxidized S units **(Sʹ)** (% S)	6	10	18	35
S/G ratio	3.4	4.3	4.9	14.1
***Chains forming inter-unit linkages and end***-***groups***				
β-*O*-4ʹ Alkyl-aryl ethers **(A)** (% S + G)	58 (82)	61 (84)	56 (85)	49 (92)
Phenylcoumarans **(B)** (% S + G)	2 (3)	1 (1)	1 (2)	0
Resinols **(C)** (% S + G)	9 (13)	8 (11)	6 (9)	2 (4)
Spirodienones **(D)** (% S + G)	2 (3)	2 (3)	2 (3)	1 (2)
Cinnamyl end-groups (**I**) (% S + G)	1 (1)	1 (1)	1 (1)	0 (1)
Total (% S + G)	71 (100)	73 (100)	66 (100)	53 (100)
C_α_-oxidized β-*O*-4ʹ ethers **(A**_**ox**_**)** (% A)	2	2	5	14

The lignin composition (S and G units), the amount of C_α_-oxidized S units (with respect to total S units), the S/G ratio, the abundance of side chains forming different inter-unit linkages (A*-*D) and cinnamyl end-groups (I) per 100 phenylpropane units, and the relative abundance of C_α_-oxidized β-O-4ʹ ethers (with respect to total β-O-4ʹ ethers) were determined from the heteronuclear single quantum correlation spectra of cellulolytic enzyme lignins isolated from the eucalypt wood treated with laccase (50 U · g^-1^) and 3% methyl syringate and laccase alone, compared with a control without enzyme and the initial wood. The percentage of side chains forming each linkage type (or end-group) are also indicated (parentheses).

The HSQC spectrum of the CEL preparation isolated from the initial eucalypt sample (Figure 3A) showed the same side-chain signals observed in the spectrum of the whole eucalypt sample (Figure 1A). These corresponded to β-*O*-4' alkyl-aryl ether (A) and resinol (C) correlations, although were better resolved and with higher intensity, and several new signals that could not be observed in the wood spectra. The latter included: spirodienone (β-1'/α-O-α') substructures (D) with their C_α_-H_α_, C_α'_-H_α',_ C_β_-H_β_ and C_β'_-H_β'_ correlations (D_α_, D_α',_ D_β,_ D_β'_); small signals corresponding to phenylcoumaran (β-5') substructures (B) with their C_α_-H_α_, C_β_-H_β_ and C_γ_-H_γ_ correlations (B_α_, B_β_ and B_γ_; the two latter correlations overlapping with other signals); a signal of cinnamyl alcohol end-groups (I) with its C_γ_-H_γ_ correlation; and signals of C_β_-H_β_ correlations in C_α_-oxidized β-*O*-4' alkyl-aryl ether substructures (A_oxβ_) (Figure 2 and Table 5). The main signals in the aromatic region of the spectrum of the initial eucalypt CEL sample corresponded to the benzenic rings of the S and G lignin units as shown for the whole wood spectrum. Signals from C_α_-oxidized S-lignin units (S'_2,6_) were also observed. Some new signals not observed in the wood spectrum appeared in this region corresponding to the above-mentioned spirodienone substructure (D) with C_2'_-H_2'_ and C_6'_-H_6'_ correlations (D_2'_ and D_6'_).

In the case of CEL preparations, the HSQC spectrum of the control sample (Figure 3B) was very similar to that of the initial material described above, although with less intense carbohydrate signals (due to the lower polysaccharide content of the control wood sample) and a slightly higher intensity of some lignin signals (Table 5).

The HSQC spectra of isolated lignins from the eucalypt samples after enzymatic pretreatment are shown in Figure 3C, D. The main differences in lignin G units and inter-unit linkages, compared with the previous samples, are shown in Table 5. Concerning lignin composition, the most noticeable effect of the enzymatic treatments of the residual lignin remaining in wood was the significant reduction in G units produced by the laccase-mediator treatment (Figure 3D), resulting in an increase of the S/G ratio from 4.3 to 14.1. Additionally, a strong increase in C_α_-oxidized S units was produced, as shown in Table 5 where the contribution of methyl syringate to the lignin S'_2,6_ signal at 106/7.3 ppm was deduced. The increase of the S'_2,6_ aromatic signal was accompanied by an increase in the C_β_-H_β_ correlations signal from β-*O*-4' ether linked C_α_-oxidized side chains (A_oxβ_). Moreover, a significant decrease in β-*O*-4' alkyl-aryl ether (A) and resinol substructures (C) per 100 phenylpropane units was the main effect observed in the side-chain region of the HSQC spectra of the lignin isolated from the wood treated with laccase and methyl syringate with the laccase-mediator treatment, together with a decrease in the less intense signals of phenylcoumarans, spirodienones and cinnamyl end-groups.

The 2D NMR analysis of lignin isolated from eucalypt samples pretreated with laccase alone (Figure 3C) also revealed some differences with respect to the control. Interestingly, the most remarkable effect was the increase of oxidized lignin structures evidenced by both aromatic (S'_2,6_) and aliphatic side-chains (A_oxβ_) signals (Table 5), revealing that laccase alone attacks lignin by an oxidative mechanism similar to that of the laccase-mediator system.

## Discussion

This work shows the potential of an oxidative enzymatic pretreatment to delignify a hardwood feedstock (*E. globulus* wood) and improve its enzymatic saccharification. Eucalypt is a rapidly growing and high biomass-producing tree used as a raw material for paper pulp manufacturing in several countries, including southwest Europe, Brazil and South Africa. Among the different eucalypt species, wood from *E. globulus* is the best raw material for kraft pulp manufacturing due to the high pulp yield [32]. Additionally, the lignin of *E. globulus* is enriched in S units with β-*O*-4' linkages predominating [32-34], which implies principally linear chains with less cross-linking than G-rich lignin because of the methoxylated and, therefore, blocked C-5 position in the S units. With all these characteristics, *E. globulus* wood has great potential as a lignocellulosic feedstock for the production of second-generation wood-based bioethanol [35].

### Delignification and improved saccharification by laccase (and methyl syringate)

Because carbohydrates in intact wood are not amenable to enzymatic hydrolysis, the use of some type of pretreatment is needed for the production of bioethanol [11,12]. To overcome the recalcitrance of lignocellulosic biomass associated with lignin, a delignification strategy based on the use of the laccase from the ascomycete *M. thermophila* and the phenolic mediator methyl syringate is reported here. Lignin removal of up to 50% from *E. globulus* wood was attained using this laccase-mediator pretreatment (with 50 U · g^-1^ of laccase and 3% of methyl syringate) followed by an alkaline peroxide extraction in a multistage sequence. The enzymatic pretreatments using laccase alone (without mediator) removed 20% of lignin from eucalypt samples (compared with 5% delignification in the control sample). This result suggests the involvement of natural phenolic structures mediating the enzymatic oxidation.

No significant decrease in the lignin content had been shown to date after laccase (alone) treatment of lignocellulosic feedstocks, such as steam-pretreated giant reed and Northern spruce [36]. Likewise, no substantial variation in lignin content and composition was reported after laccase-mediator treatment of steam-exploded eucalypt wood with *M. thermophila* laccase (Novozym 51003 from Novozymes) [37]. This is most probably due to the different mediator, HBT, which is scarcely oxidized by *M. thermophila* laccase [38], and treatment conditions, although the modified lignin structure after steam explosion could also have some influence. *M. thermophila* and other ascomycete laccases have lower redox potential than basidiomycete laccases [39]; however, this is not a problem when an easily oxidized phenolic mediator is used, as shown for paper pulp bleaching [29]. In return, ascomycete enzymes are more easily over-expressed in industrial hosts as recombinant proteins, enabling their commercialization for industrial applications (as in the case of *M. thermophila* laccase produced by Novozymes).

Enzymatic removal of lignin from ground eucalypt wood had been recently reported by Gutiérrez *et al*. [24] using the high redox-potential laccase from *Trametes villosa* and HBT as mediator. However, the cost, safety and environmental profile of this synthetic mediator make its implementation at an industrial scale difficult. To overcome these limitations, several lignin-derived phenols selected as natural laccase mediators [40] have been investigated for paper pulp biobleaching [27-29]. Our results showing methyl syringate helping laccase to delignify eucalypt wood are in agreement with laccase oxidation of non-phenolic model compounds in the presence of this and related phenolic mediators [41]. The present paper shows the potential of using a commercial laccase and a natural phenol as a mediator for delignifying a woody feedstock. The enzyme used is the thermostable laccase from the ascomycete *M. thermophila*, which has been cloned, expressed in *Aspergillus oryzae*, biochemically characterized, improved for different applications, and commercialized [39,42]. The mediator used, methyl syringate, is a natural product and shows promising results as a laccase mediator due to a suitable redox potential [43]. The above combination (of commercial enzyme and low-cost mediator) facilitates industrial feasibility of the laccase-mediator pretreatment.

As expected from the decrease of (Klason) lignin content, the pretreatment of eucalypt wood with the *M. thermophila* laccase and methyl syringate improved the saccharification yield similarly (about 40%) with the two different doses of laccase (10 U · g^-1^ and 50 U · g^-1^) and mediator (1% and 3%, respectively) used. An improvement on cellulose hydrolysis of ensiled corn stover was attained by the use of laccase from *Trametes versicolor* and HBT using higher doses of laccase (4,000 U · g^-1^) and mediator (5%) and cellulolytic enzymes (15 FPU · g^-1^ cellulase, and 1,000 nkat · g^-1^ β-glucosidase) [25]. Likewise, an improvement in cellulose hydrolysis was reported when treating steam-pretreated softwood with *Trametes hirsuta* laccase and the mediator *N*-hydroxy-*N*-phenylacetamide [23]. The relatively low doses of cellulases used in the present work (2 FPU · g^1^ Celluclast 1.5 L and 100 nkat · g^-1^ of β-glucosidase) to obtain a glucose yield of 55% are noteworthy. As a significant decrease in the cost of cellulases is necessary for the economic conversion of lignocellulose to ethanol [44], high hydrolysis yields at low dosage of cellulases are highly desirable.

### Structural modification of lignin by laccase (and methyl syringate)

During wood delignification by laccase and methyl syringate, an important fraction of the lignin is depolymerized and released from the sample causing the 50% reduction of Klason lignin. However, 2D NMR and other analyses of the whole wood and its isolated CEL revealed that the residual lignin remaining in wood is also modified during the enzymatic pretreatment.

The general structure of the eucalypt lignin, in terms of aromatic units and inter-unit linkages, agrees with that reported in previous studies [30,32-34]. The most important modification of the residual lignin during wood pretreatment with laccase and methyl syringate was the strong increase in C_α_-oxidized S units, revealed by 2D NMR analyses of the whole wood and the CEL preparations. Another noticeable effect shown by the 2D NMR analyses, and also by Py-GC/MS, was the relative reduction in G lignin units produced by the laccase-mediator treatment, resulting in a strong increase of the S/G ratio (lignin composition by 2D NMR also includes condensed structures that are recalcitrant towards pyrolytic breakdown). The decrease in lignin G units, which occurred to a greater extent than that of the S lignin units, has previously been observed in the pretreatment of eucalypt wood with *T. villosa* laccase and HBT [24]. Moreover, a significant decrease in the number of side-chains involved in the different lignin substructures (per 100 phenylpropane units) was observed after the laccase treatment in the presence of methyl syringate, in agreement with progressive depolymerization.

Generation of oxidized lignin structures is congruent with the nature of the lignin biodegradation process, which has been described as an ‘enzymatic combustion’ where fungal oxidoreductases play a central role [45]. It is generally accepted that lignin degradation by white rot fungi and their ligninolytic peroxidases starts by aromatic ring oxidation to a cation radical, but quickly leads to side-chain C_α_-C_β_ cleavage, causing depolymerization [46]. The same mechanism has been suggested for some laccase reactions mediated by synthetic compounds, for example 2,2-azinobis(3-ethylbenzothiazoline-6-sulfonate), (ABTS), but the action of laccase-HBT on non-phenolic lignin models is predominantly produced by hydrogen-atom abstraction from the C_α_ position, followed by alkyl-aryl ether breakdown [47,48].

The above attack mechanism would result in the increased amount of C_α_-oxidized S lignin units found in eucalypt wood pretreated with *T. villosa* laccase and HBT [24]. The presence of oxidized S lignin units was also observed in eucalypt pulp residual lignin after laccase-HBT treatment, including both C_α_ ketones and carboxylic acids [49]. Additionally, lignin side-chain oxidation by laccase-HBT has been reported after Py-GC/MS and thermochemolysis of laccase-treated eucalypt wood and corn stover, respectively [25,50]. Wood lignin modification by laccase in the presence of methyl syringate also yielded a structural modification pattern characterized by extensive C_α_-oxidation (as shown by 2D NMR), suggesting that the attack mechanism by laccase in the presence of methyl syringate is the same reported by laccase-HBT. This agrees with results from model compounds showing that laccase in the presence of phenolic mediators oxidizes non-phenolic aromatic compounds via a hydrogen abstraction mechanism [51].

## Conclusions

Eucalypt feedstock can be delignified by a high-yield expressed (recombinant) laccase when applied in a sequence consisting of successive enzymatic and alkaline peroxide extraction stages, directly on the ground lignocellulosic material (that is, without a previous chemical deconstruction pretreatment). Lignin removal reached 50% when methyl syringate, a natural and potentially cheap mediator, was applied together with the enzyme. The pretreated eucalypt feedstock was hydrolyzed with higher efficiency than the untreated material, releasing higher yields of glucose and xylose using relatively low doses of cellulases. Preferential removal of lignin G units, in comparison to S units, and breakdown of main inter-unit linkages was suggested by Py-GC/MS, and confirmed by 2D NMR. The 2D NMR spectra of whole wood (at the gel stage) also showed: the selective action of laccase-mediator on the lignin moiety, while the polysaccharide signals remained unchanged with respect to the controls; and the extensive presence of oxidized S units in the residual lignin remaining in pretreated wood. These and other changes in lignin structure were analyzed in depth by 2D NMR of isolated lignins. The above results provide evidence for a C_α_-oxidation mechanism of lignin degradation even on treatment with laccase alone.

## Methods

### Wood, enzyme and mediator

Eucalypt (*E. globulus*) wood chips from ENCE (Pontevedra, Spain) were air-dried and ground in an IKA MF10 cutting mill to pass through a 100-mesh screen, and then finely milled using a Retsch PM100 planetary mill (Retsch, Haan, Germany) at 400 rev · min^-1^ (with 5 min breaks after every 5 min of milling) using a 500 mL agate jar and agate ball bearings (20 × 20 mm). The total ball-milling time for the samples was 5 h.

A commercial (recombinant) fungal laccase from the ascomycete *M. thermophila*, provided by Novozymes (Bagsvaerd, Denmark), was used in this study. Its activity was measured as initial velocity during oxidation of 5 mM ABTS from Roche to its cation radical (ϵ_436_ 29300 M^-1^ · cm^-1^) in 0.1 M sodium acetate (pH 5) at 24°C. The laccase activity of the enzyme preparation was 945 U/mL. One activity unit (U) was defined as the amount of enzyme transforming 1 μmol of ABTS per min.

Methyl syringate (methyl 4-hydroxy-3,5-dimethoxybenzoate) from Alfa Aesar (Karlsruhe, Germany) was used as the mediator.

### Laccase-mediator treatments

The eucalypt samples were treated with the *M. thermophila* laccase in the presence (and absence) of the mediator methyl syringate. Laccase doses of 10 U · g^-1^ and 50 U · g^-1^were assayed, together with 1% and 3% methyl syringate, respectively (doses refer to wood dry weight). The treatments were carried out in 200 mL pressurized bioreactors (Labomat, Mathis, Oberhasli/Zürich, Switzerland) placed in a thermostatic shaker at 170 rev · min^-1^ and 50°C, using 10 g (dry weight) samples at 6% consistency (w:w) in 50 mM sodium dihydrogen phosphate (pH 6.5) under O_2_ atmosphere (2 bars) for 24 h. After the treatment, the samples were filtered through a Büchner funnel and washed with 1 L of water. In a subsequent step, samples at 6% consistency (w:w) were submitted to a peroxide-reinforced alkaline extraction using 1% (w:w) sodium hydroxide and 3% (w:w) hydrogen peroxide (also with respect to sample dry weight) at 80°C for 90 min, followed by water washing [29]. Cycles of four successive enzyme-extraction treatments were applied. Treatments with laccase (10 U · g^-1^ and 50 U · g^-1^) alone (without mediator) and controls without laccase and mediator were also performed (followed in both cases by the corresponding alkaline extractions). A control with mediator alone was not included, taking into account the results from previous studies. Klason lignin content was estimated according to TAPPI Method T222 om-88 [52].

### Saccharification of treated wood

The laccase-pretreated samples were hydrolyzed with a cocktail containing commercial enzymes (from Novozymes, Bagsvaerd) with cellulase (Celluclast 1.5 L; 2 FPU · g^-1^) and β-glucosidase (Novozym 188; 100 nkat · g^-1^) activities, at 1% consistency in 3 mL of 100 mM sodium citrate (pH 5) for 72 h at 45°C, in a thermostatic shaker at 170 rev · min^-1^ (in triplicate experiments).

The different monosaccharides released were determined as alditol acetates [53] by GC. An HP 5890 gas chromatograph (Hewlett-Packard, Hoofddorp, The Netherlands) equipped with a split-splitless injector and a flame ionization detector was used. The injector and detector temperatures were set at 225°C and 250°C, respectively. Samples were injected in the split mode (split ratio 10:1). Helium was used as the carrier gas. The capillary column used was a DB-225 (30 m × 0.25 mm internal diameter, 0.15 μm film thickness) from Agilent J&W (Folsom, CA). The oven was temperature-programmed from 220°C (held for 5 min) to 230°C (held for 5 min) at 2°C min^-1^. Peaks were quantified by area; glucose, xylose and arabinose were used as standards to elaborate calibration curves. The data from the three replicates were averaged.

### Enzymatic isolation of lignin

The air-dried eucalypt samples were extracted three times with water then three times with 80% ethanol by sonicating in an ultrasonic bath for 30 min each time. CEL preparations were isolated by enzymatically saccharifying polysaccharides as described by Chang *et al*. (1975) [54]. Cellulysin cellulase (Calbiochem), a crude cellulase preparation from *Trichoderma viride* also containing hemicellulase activities, was used. Its activity was ≥10,000 FPU · g^-1^ of dry weight. The extractives-free ball-milled material (200 mg) was suspended in 30 mL of 20 mM sodium acetate (pH 5.0) in a 50 mL centrifuge tube, 7.5 mg of Cellulysin was added, and the reaction slurry was incubated at 30°C for 48 h. The solids were pelleted by centrifugation (8,000 rpm, 4°C, 20 min), and the process was repeated with fresh buffer and enzyme, three times. Finally, the residue (CEL) was washed with distillated water, recovered by centrifugation and freeze dried.

### Pyrolysis-gas chromatography/mass spectrometry

Pyrolysis of eucalypt wood samples (approximately 100 μg) was performed with an EGA/PY-3030D micro-furnace pyrolyzer (Frontier Laboratories Ltd., Fukushima, Japan) connected to an Agilent 7820A gas chromatograph using a DB-1701 fused-silica capillary column (60 m × 0.25 mm internal diameter, 0.25 μm film thickness) and an Agilent 5975 mass selective detector (EI at 70 eV). The pyrolysis was performed at 500°C. The oven temperature was programmed from 45°C (4 min) to 280°C (10 min) at 4°C min^-1^. Helium was the carrier gas (1 mL min^-1^). The compounds were identified by comparing their mass spectra with those of the Wiley and NIST libraries and reported in the literature [55,56]. Peak molar areas were calculated for the lignin-degradation products, the summed areas were normalized, and the data for three repetitive analyses were averaged and expressed as percentages. The relative standard deviation for the pyrolysis data was less than 5%.

### Two-dimensional nuclear magnetic resonance spectroscopy

For gel-state NMR experiments, approximately 100 mg of finely divided (ball-milled) extractive-free wood samples were directly transferred into 5 mm NMR tubes, and swelled in 1 mL of DMSO-*d*_*6*_, forming a gel inside the NMR tube [30,31]. For a more in-depth structural characterization of the lignins, around 30 mg of CEL preparations were dissolved in 0.75 mL of DMSO-*d*_*6*_.

HSQC 2D-NMR spectra were acquired at 25°C on a Bruker AVANCE III 500 MHz spectrometer (Bruker Biospin, Fallanden, Switzerland) fitted with a cryogenically cooled 5 mm TCI gradient probe with inverse geometry (proton coils closest to the sample). The 2D ^13^C-^1^H correlation spectra were carried out using an adiabatic HSQC pulse program (Bruker standard pulse sequence 'hsqcetgpsisp2.2') and the following parameters: spectra were acquired from 10 to 0 ppm (5,000 Hz) in F2 (^1^H) using 1,000 data points for an acquisition time of 100 ms, an interscan delay of 1 s, and from 200 to 0 ppm (25,168) in F1 (^13^C) using 256 increments of 32 scan, for a total acquisition time of 2 h 34 min. The ^1^*J*_CH_ used was 145 Hz. Processing used typical matched Gaussian apodization in ^1^H and a squared cosine bell in ^13^C. The central solvent peak was used as an internal reference (δ_C_/δ_H_ 39.5/2.49). The ^13^C-^1^H correlation signals from the aromatic region of the spectrum were used to estimate the lignin composition in terms of G, S and oxidized S (S') units, and those of the aliphatic-oxygenated region were used to estimate the inter-unit linkage and end-unit abundances. The quantification was carried out using correction factors based on estimated carbon-proton coupling constants. The S lignin content in the laccase-mediator-treated sample was corrected for the contribution of methyl syringate to the 106/7.3 ppm signal, which was estimated from the integral of its characteristic signal at 52/3.8 ppm.

## Abbreviations

CEL: Cellulolytic enzyme lignin; DMSO-d6: Deuterated dimethylsufoxide; FPU: Filter-paper units; G: Guaiacyl; HBT: 1-hydroxybenzotriazole; HSQC: Heteronuclear single-quantum correlation; NMR: Nuclear magnetic resonance; Py-GC/MS: Pyrolysis gas chromatography/mass spectrometry; S: Syringyl.

## Competing interests

The authors declare that they have no competing interests.

## Authors’ contributions

AR carried out all of the experimental work presented here, including the enzymatic pretreatments, saccharification assays and GC analyses and lignin isolation. JR conducted the 2D NMR and Py-GC/MS analyses including data interpretation. JCR contributed in pyrolysis data interpretation. ATM substantially contributed to the 2D NMR data interpretation and quantification and was involved in critically reviewing the manuscript with substantial contribution to its intellectual content. AG conceived of the study, supervised the work, substantially contributed to analysis and interpretation of data and wrote the manuscript. All authors read and approved the final manuscript.
